# Dental Caries and Its Management

**DOI:** 10.1155/2023/9365845

**Published:** 2023-01-03

**Authors:** Abdulhadi Warreth

**Affiliations:** Restorative Department, College of Dentistry, Ajman University, Ajman, UAE

## Abstract

**Objectives:**

In recent years, the management of dental caries has evolved significantly. Caries prevention, early detection, and a diagnosis based on risk indicators and risk factor assessments are the most current practical approaches. Furthermore, as proposed in minimally invasive dentistry, the new management approaches preserve healthy tissue and maintain pulp vitality. This article overviews the latest minimally invasive dental caries management and treatment options. The information will assist the reader in the early detection, diagnosis, and treatment of dental caries.

**Materials and Methods:**

The PubMed (MEDLINE) search engine was used to gather the most relevant information on dental caries. The search was restricted to five years (May 30, 2018–May 29, 2022), and only English-language studies were accessed. A Boolean search of the PubMed data set was implemented to combine a range of keywords. The following filters were applied: abstract, free full text, full text, clinical trial, randomised control trial, systematic review, meta-analysis, and review. More studies were also obtained by manual searches from Google Scholar and textbooks on dental caries.

**Results:**

By using this process, 683 articles and studies were obtained. The most relevant published studies were chosen and used in the current review. The selected articles are included in the references list. However, the search extended to cover the last five years as our understanding and management of dental caries have changed significantly.

**Conclusions:**

Early detection and diagnosis of caries based on risk indicators and risk factor assessments are effective. Furthermore, minimally invasive restorative techniques are beneficial in managing dental caries and preserving healthy tissue and should be used whenever possible. This new information, knowledge, and materials should encourage professionals to implement this method. Having a strategy and system based on patient-centred care is critical, and our dental responsibilities must prioritise patient-centred care.

## 1. Introduction

Management of dental caries has changed significantly in recent years [1–3]. The most contemporary practical approaches are based on early caries detection and prevention. They are also built on making a diagnosis based on risk indicators and risk factor assessment [1, 3, 4].

The new management approaches aim to preserve healthy tissue, as proposed in minimally invasive dentistry [1, 2, 5]. This aims to achieve several goals, such as the implementation of a preventive philosophy, individualised risk assessments for patients, early detection of carious lesions, and remineralization of the noncavitated lesion [2, 3].

Restorative procedures are damaging to tooth tissue and may endanger the tooth in the long term when it enters the restoration-rerestoration cycle [4]. Therefore, when restorative intervention is needed, the procedure used should be as minimally invasive as possible [3, 5]. This includes repairing, refurbishing, or polishing rather than replacing defective restorations [1, 6, 7].

Nevertheless, when the pulp is exposed by caries, it can be managed in a more conservative way than that previously used. This includes using vital pulp treatment (VPT) such as partial or complete pulpotomy instead of pulpectomy [2, 8].

Unfortunately, many dentists continue to treat dental caries and pulp disease with invasive procedures [9]. However, it will undoubtedly take time for a shift to noninvasive and minimally invasive approaches in everyday clinical practice [5, 10–12].

This article provides an overview of minimally invasive dental caries control. It also discusses the different procedures used to accomplish minimally invasive dentistry based on the extension of the carious lesion. This information will assist the reader in detecting, diagnosing, and treating dental caries in its early stages as well as when it reaches the dentine, utilising minimally invasive treatment options.

## 2. Materials and Methods (Searching Procedure)

### 2.1. Search Strategy

The MEDLINE [13] database through PubMed was used to identify papers containing dental caries and associated definitions, epidemiological considerations, aetiological agents, and risk factors. A Boolean search was used to combine a variety of terms. The following filters were also used: abstract, free full text, full text, clinical trial, randomised controlled trial, systematic review, meta-analysis, and review. The search was restricted to a five-year period (May 30, 2018–May 29, 2022) and only English-language studies. The most relevant published studies that met the set inclusion criteria were chosen and used in the current review.

### 2.2. The Inclusion and Exclusion Criteria

Studies on dental caries and caries lesions published in English-language in the last five years period (May 30, 2018–May 29, 2022) were selected. These included:

(i) In vivo studies (prospective and retrospective)
(ii) In vitro studies on the histology of dental caries
(iii) Data on dental caries and caries lesions obtained from
  - Peer-reviewed articles
  - Systematic review and meta-analysis
  - Google Scholar
  - Recently published textbooks
  - Manual searches from the reference list of selected articles.

Exclusion criteria included studies that did not meet the inclusion as mentioned above criteria.

## 3. Results

Six hundred and eighty three articles and studies were obtained. The references list includes the articles and studies used in this review.

### 3.1. Dental Caries

Dental caries is a complex multifactorial disease characterized by demineralisation of dental hard tissue (enamel, dentine, and cementum) in deciduous and permanent teeth [1, 3, 4, 7, 14–16]. If properly managed, dental caries is a preventable and a reversible disease [14].

Four elements are necessary for the development of dental caries. These elements are bacterial biofilm (plaque), fermentable carbohydrates, dental hard tissue, and time [17]. Furthermore, personal and oral environmental factors have a substantial impact on the onset and course of the disease [3, 14].

### 3.2. Examination, Detection, and Diagnosis of Dental Caries

#### 3.2.1. Visual-Tactile and Radiographic Examination of Dental Caries

The diagnosis of dental caries is based on the clinical examination, commonly carried out by a visual-tactile method [18–20], which is frequently supported by a radiograph [21]. Furthermore, fibre-optic transillumination (FOTI), electrical conductivity, and laser fluorescence are frequently used to diagnose dental caries [22].

**Figure 1:**
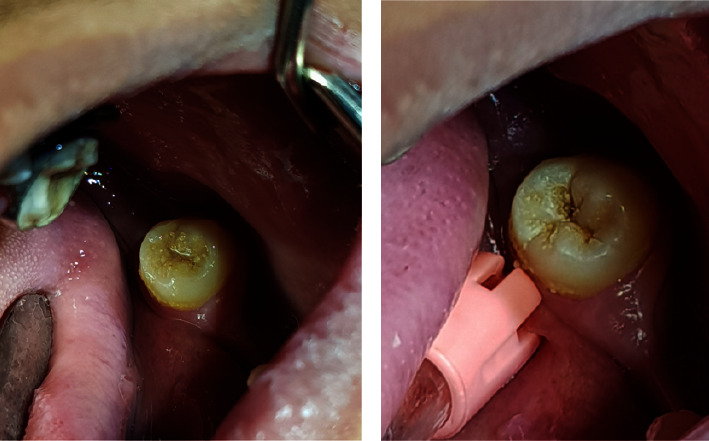
Teeth should be cleaned to remove food debris that may conceal dental caries. A carious lesion is covered by food debris in (a), and it is visible after cleaning (b).

The visual examination requires good lighting and a clean, dry tooth [23]. It is also essential to clean the tooth surface before the examination [24] (Figure 1).

Traditionally, the dental explorer is used clinically in detecting carious lesions. However, its use is controversial and debatable because it provides no further benefits [25, 26]. For instance, sticking the explorer in the fissure does not necessarily indicate caries exists but may indicate that the explorer fits snugly in the fissure. Moreover, the explorer may cause irreversible physical damage to the demineralized fragile surfaces [4, 25, 27]. The use of the explorer may also lead to spreading cariogenic bacteria deeper and can settle in areas where routine oral hygiene procedures are ineffective. A dental explorer can also transmit cariogenic bacteria from infected to noninfected pits and fissures [28]. Furthermore, using the explorer has low sensitivity values [14, 28].

#### 3.2.2. Radiographic Assessment

The visual inspection combined with a radiographic examination is routinely used to examine and diagnose occlusal and proximal caries [19, 21, 26]. However, it should be remembered that radiography is 2-dimensional imaging of a 3-dimensional object and that at least 25% of the mineral content of tooth structure has to be lost before dental caries can radiographically be seen [29]. For instance, cavitation is unlikely when interproximal carious lesions are radiographically confined to the enamel. Carious lesions, on the other hand, are more likely to cavitate if they reach the middle third of the dentine. Lesions that reach the dentine's outer third or are located around the dentin-enamel junction are more likely to vary [12].

We should be aware that radiographs may underestimate the extension of the carious lesion as it is more likely to be deeper than its radiographic image [26]. The interpretation of radiographs should be carried out with caution, as distinguishing between artefacts such as burn-out or Mach bands and proximal caries is challenging. Furthermore, overlapping, overexposure, and underexposure may negatively affect the images of the carious lesion.

When a dental radiograph is required, its use must be optimized with the goal of limiting the patient's exposure to ionizing radiation following the ALADAIP principle (As Low as Diagnostically Achievable being Indication-oriented and Patient-specific). The alternative, such as fibre-optic transillumination, should always be considered [30, 31]. The transillumination technique can be used to detect proximal carious lesions. However, unlike bitewing radiographs, transillumination cannot be used to monitor dental caries [21].

#### 3.2.3. Histological and Clinical Description of Carious Lesions

**Figure 2:**
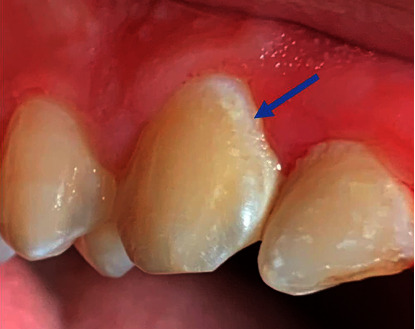
A clinical photograph of a white spot lesion on the canine cervical region (the blue arrow).

*(1) Carious Lesions in Enamel (White Spot Lesion).* The first stage of enamel caries is observed as a demineralisation area seen clinically as a white spot lesion (WSL) [32, 33]. The WSL is also known as an initial or early carious lesion. It appears clinically as a chalky white zone when the tooth surface is dry (Figure 2). The high porosity in the subsurface zone due to demineralisation gives the WSL this distinct appearance [29]. In contrast to the subsurface, the surface is relatively intact, with a small number of pores. The contents of the pores (saliva or air) alter light transmission due to differences in the refractive indices of enamel, air, and saliva. Air, water, and enamel have refractive indices of 1.00*n*, 1.33*n*, and 1.66*n*, respectively. As the difference in refractive indices between enamel and air is about 0.66*n*, caries is more visible when the saliva in the enamel pores is replaced with air when the tooth is dry. As a result, the tooth surface and suspected lesion must be dry, allowing water in the pores to be replaced with air, allowing WSL to be seen clearly. Figure 2 represents clinical images of WSLs.

WSL is also common around fixed orthodontic appliances and is considered a significant challenge during fixed orthodontic treatment [32–35].

**Figure 3:**
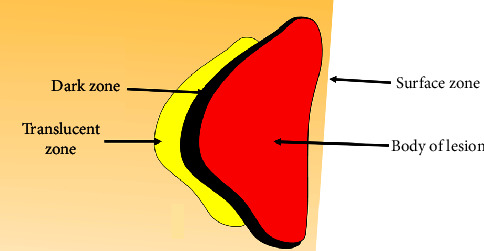
A schematic representation of histological zones of noncavitated demineralized enamel carious lesion (WSL).

The WSL is made up of four zones: the surface zone, the body lesion, the dark zone, and the transparent zone. The surface zone is relatively intact, with a pore volume of less than 1%. The body zone, which is located between the surface and the dark zone, is the most demineralized, with pore volumes ranging from 5% to 25%. The dark zone contains pore volume ranges of between 2 and 4%. The transparent zone is the innermost zone in the advancing front of the lesion and has a pore volume of around 1% and somewhat more pores than sound enamel. However, it is not always present.  Figure 3 schematically displays the demineralized enamel zones (WSL) [29].

*(2) Carious Lesions in Dentine.* Dentine and enamel have structural differences, so the progression of caries in dentine differs from that in enamel. Dentine has low minerals and contains microscopic tubules that allow bacteria to enter and minerals to exit, causing the enamel lesion's body to become increasingly demineralized. Consequently, the surface enamel weakens and eventually collapses [1, 29].

Caries dentine is histologically divided into four zones: soft (infected) dentine, firm (affected) dentine, dark zone and transparent zone. The first and second zones are more clinically relevant than the third and fourth zones. As a result, the discussion will focus on these two zones.

- Soft dentine
- Soft dentine was previously referred to as infected dentine, and it is also known as outer dentine caries. It is the most superficial, necrotic, and irreparable dentine. It is easily excavated with a hand excavator and removed with rotary tools [29]. Its mineral component is dissolved by the acid, and its collagen matrix is denatured by proteolytic enzymes [9]. Dentinal tubules are enlarged and deformed, and they contain a great number of bacteria (Table 1). Clinically, the soft dentine is seen as dark-brown, soft, moist, and mushy [4].
- The soft dentine is frequently removed during the caries removal step of cavity preparation because it is necrotic, irreparable, and cannot serve as a reliable binding substrate for the adhesive material to produce a reliable seal [1, 2, 7]. In recent years, there has been widespread agreement that soft dentine can be preserved near the pulp [1, 2].
- Firm dentine
- Firm dentine was formerly known as “affected” dentine [1]. It is also called inner carious dentine. It is firmer than the soft dentine and resistant to hand excavation because of its high mineral and collagen contents (Table 2). Its mineral dissolution is lesser than that of the soft dentine. However, firm dentine is considered partially demineralized dentine. It is a paler brown, sticky, harder, and scratchy to a sharp probe [19]. Firm dentine can be found directly beneath soft dentine. It is slightly demineralized, yet it is still capable of remineralization and recalcification. Continuous mineral deposition within the tubules beneath a carious lesion process causes tubular obliteration and sclerosis, potentially lowering bond strengths.
- The collagen cross-linking remains intact and can serve as a template for remineralization of intertubular dentine. As a result, if the pulp remains viable, firm dentine can be remineralized.
- Hard dentine
- It includes normal (sound), tertiary, and sclerotic dentine. Clinically, it cannot be easily penetrated with a blunt explorer and can only be removed by a bur or a sharp cutting instrument.

The dentine-pulp complex responses to thermal, chemical, bacterial, or mechanical stimulation, caries, and fracture lead to tertiary dentine formation. The tertiary dentine can be classified into reactionary and reparative dentine [36]. The former results from a mild stimulus, such as normal tooth wear and the latter is due to an extensive injury, like caries/cavity preparation [37].

**Figure 4:**
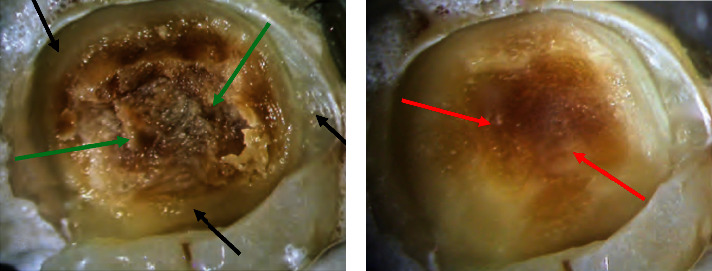
Nonselective caries removal to hard dentine at the periphery (black arrows) (a). Green arrows indicate soft dentine, (b) selective caries removal to firm (affected) dentine overlying the pulpal aspect (red arrows).

Reactionary dentine is formed by odontoblasts in the pulp chamber wall near the area corresponding to the damage caused by factors such as carieses. In the event of pulp exposure, newly differentiated odontoblast-like or odontoblastoid cells replace irreversibly injured odontoblasts at the exposure site, forming a reparative a tubular dentine bridge [29, 38].  Figure 4 displays firm and hard dentine.

## 4. Caries Removal Strategies

In general, there are two approaches to caries removal of cavitated carious lesions in teeth with sensible and asymptomatic pulps: nonselective caries removal and selective caries removal [3, 5, 7, 39].

- The nonselective caries removal (complete caries removal) approach
-It is the traditional method of treating dental caries [3, 40]. It represents the removal of both soft and firm dentine, regardless of the closeness of the carious lesion to the pulp. It is also known as complete caries removal or complete caries excavation. It can also be denoted as caries removal to hard dentine. This method removes caries dentine; soft and firm caries regardless of depth or proximity to the pulp. This approach's rationale is that caries is prevented from spreading further as all bacteria and caries are eradicated [1, 7, 41]. Furthermore, restorative materials can be effectively placed and retained because a strong basis is available by providing hard sound dentine [1, 3] However, it is associated with a high rate of pulp exposure [1, 3, 7, 41]. This method is deemed nonconservative and excessive, and its validity is being questioned. This is because there are no evidence-based arguments to justify its use [1, 3]. Nevertheless, it may be implemented when the carious lesions are not close to the pulp.
- The selective caries removal approach
- In this method, caries are selectively removed according to their proximity to the pulp, and therefore, soft and/or firm dentine is left and preserved. This approach is also known as the partial caries removal (PCR) method [3, 5, 7, 39]. This approach is categorised into a one- or two-step method [1]. In the one-step method, the caries dentine is selectively removed, and the cavity is restored with a permanent restoration in a single visit. Indirect pulp capping (IPC) is an example of a one-step method of this approach. In contrast, the two-step caries removal technique, such as the stepwise (SW) method, involves removing carious dentine in two different clinical appointments [42].

The reasons for using the selective caries removal methods are to avoid pulp exposure and maintain the pulp vitality. According to Bjørndal et al. [43], the pulp exposure rate in the SW method was significantly lower than in the nonselective caries (complete) removal to hard dentine (21.2% versus 35.5%). The differences were statistically significant (*P*=0.014) [43].

Supporters of the one-step selective caries removal technique argue that re-entry, advocated for use in the two-step (SW method) technique, is unnecessary as it may endanger the pulp and lead to its exposure [3, 7, 44]. On the other hand, supporters of the two-step procedure debate that the soft dentine left in the one-step technique will shrink, resulting in a defective permanent restoration [1].

The selective caries removal method is further divided into two subcategories based on the type of caries dentine removed: selective caries removal to soft dentine and selective caries removal to firm dentine. While the soft dentine is preserved in the first method, the soft dentine is removed, but the firm dentine is preserved in the second.  Figure 4 shows a carious lesion before and after caries removal to hard and firm dentine. It is important to note that the terms soft and firm dentine previously denoted infected and affected dentine, respectively. These two terms are used in this article.

- Selective caries removal to soft dentine (soft dentine is retained)
- The pulpal and axial soft caries dentine is left to prevent pulp exposure and “stress” to the pulp. However, the peripheral dentine and dentine-enamel junction are carried out using rose head burs or a sharp excavator until hard, dry dentin remains (nonselective) to ensure an appropriately sealed restoration. Compared to nonselective caries removal to hard dentine, this approach dramatically minimizes the likelihood of pulpal exposure [3, 43, 45]. As a result, it is advised to use it in extremely deep cavitated carious lesions.
- Selective caries removal to firm dentine (firm dentine is retained)

This method varies from the previous approach in that soft dentine is removed from the cavity's pulpal aspect while firm dentine is retained near the pulp [2]. It is recommended for use in shallow or moderately deep cavitated dentine carious lesions to avoid pulp exposure and maintain pulp vitality. However, as previously mentioned, the cavity's boundaries should consist of only hard dentine. This approach is also indicated when lesions radiographically extend to less than the pulpal third or quarter of the dentine. Nevertheless, it should be avoided if the carious lesion is exceedingly deep and close to the pulp, as pulp exposure is inevitable [1, 3, 19].

## 5. Noncarious Removal/Caries Sealing Approach

It is indicated for a clinically noncavitated occlusal carious lesion that radiographically appears to extend into the dentine. Hence, these lesions may be sealed using fissure sealants when plaque control alone is insufficient to stop the decay [7, 41]. However, continuous monitoring is required to ensure the integrity of the sealant material and that the lesion does not progress [7, 41]. This approach may also be indicated for use in selective cavitated carious dentine lesions [30, 46]. Consequently, the carious lesion becomes inactive because it is sealed. Composite resins or glass ionomers are used as sealant materials. This method is discussed below.

## 6. Nonrestorative Cavity Control (NRCC) Approach

This method is determined by the shape and depth of the carious lesion, the patient's ability to maintain good oral hygiene and avoid plaque accumulation, and the patient's aesthetic requirements. The cavity opening is widened to make it more cleanable, easy to clean and improve patients' abilities to clean it [7, 41]. As a result, the patient cleans the teeth repeatedly to remove the biofilm to stop the progression of the lesion, remineralization therapies such as fluoride through toothbrushing is utilized [7, 47]. It is critical to change patient behaviour to control the biofilm and change the habits that led to the development of the lesion [7]. Hence, it is also crucial to educate the patient about the causes of dental caries and how they can be reduced or eliminated. This approach is also recommended in cases of early-stage active root surface caries with a shallow defect [22]. As a result, good oral hygiene and fluoride treatment can be tried first to promote remineralization and manage caries [22].

## 7. Methods of Management of Carious Lesions

Patients who have been treated for dental caries are still at risk of developing more carious lesions and disease progression in the future, if risk factors are not successfully controlled. These patients will require long-term care.

### 7.1. Caries Management by Risk Assessment [48, 49]

Prevention is always better than cure, and different phases and activities of caries may necessitate a different management method. The Caries Management by Risk Assessment (CAMBRA) system, developed in 2002, is regarded as a reliable patient-centred approach. It takes a patient's health and lifestyle risk factors into consideration. It is based on peer-reviewed publications that looked at caries risk assessment and the impact of saliva and nutrition in caries development [50]. The CAMBRA approach was evaluated and validated for usage, and it has been shown to be useful guidance to health care professionals for caries evaluation and management [48]. Accordingly, patients are divided into three groups based on their risk of developing dental caries: high, moderate, and low risk. Its adoption allows for tailored preventative counselling and action based on the individual's risk level [48]. The CAMBRA guidelines are summarized in Table 3.

Chemical therapy that represents the use of an antibacterial agent and fluoride treatment is required for high- and extreme-risk patients. To lessen the bacterial challenge, change the biofilm, and provide prevention rather than ongoing caries development, fluoride therapy must be supplemented by the use of an antibacterial agent [48]. A combination of daily antibacterial therapy (0.12% w/v chlorhexidine gluconate mouth rinse) and twice daily high concentration fluoride toothpaste (5,000 ppm fluoride), both for home use, are recommended for high-risk and extreme-risk patients [48].

In high-caries-risk adult individuals, daily usage of a combination of chemical therapy and restorative treatment was observed to decrease caries by 20–38% [48]. Topical fluoride has resulted in a considerable decrease in smooth surface caries [48].

However, according to Momoi and associates [22], cavity preparation is indicated and intervention immediately needed where more than one of these findings is evident:

1. A cavity is visually detected after cleaning and drying the tooth.
2. There is pain or discomfort from cold water or food-impaction.
3. There is unacceptable appearance.
4. Radiographs reveal carious lesions penetrating more than a third of the dentin.
5. A patient is at high risk of caries.

### 7.2. White Spot Lesions and Their Management (WSLs)

Recently, there has been widespread agreement that sealing noncavitated carious lesions is a viable option for controlling the carious lesion even if they have reached the outer dentine surface. This is one of the most conservative approaches to preserving tooth structure and pulp vitality while avoiding invasive treatment. The method is referred to as the “microinvasive concept” [51].

WSL can be treated noninvasively, as a result, with good oral hygiene, the use of fluoride-containing toothpaste, mouthwash, gels, and varnish, casein phosphopeptide amorphous calcium phosphate (CPP-ACP) and casein phosphopeptide-amorphous calcium phosphate fluoride (CPP-AFCP), are all advised [30, 34, 52–54]. It can also be managed using the resin infiltration technique, which has been shown to delay or reverse the progression of noncavitated carious lesions [55]. This method is discussed in more detail below.

### 7.3. Sealing of Noncavitated Caries Lesions

As previously mentioned, sealing the noncavitated carious lesion has been shown to stop lesion progression in vivo and in vitro effectively [51, 56–58]. A proximal carious lesion can be sealed after separating the affected tooth to reach the lesion. Resin infiltration and sealing were more effective than noninvasive treatments (e.g., fluoride varnish) for halting noncavitated proximal lesions, according to Chen et al. [51].

The material used as a sealer has a significant effect on the efficiency of the sealing outcome. For instance, according to Arslan et al. [57], selection of resin type is critical as certain resins may be affected by water sorption over time than other resin materials. As a result, this approach must be used with extreme caution. Care must be taken in case selection, application, and follow-up. However, more long-term randomised clinical trials are needed to contribute to this body of evidence [59].

Sealing and resin infiltration of the carious lesion are two microinvasive approaches. Both involve the removal of the dental hard tissue surface at the micron level, typically performed during an etching step, such as in sealing or infiltration techniques [12]. The infiltration techniques involve etching with an acid such as 15% HCl-gel for a specific time, such as 120 seconds, followed by an infiltrating resin (“Resin Infiltration”; Icon; DMG) [11, 12, 60].  Table 4 shows the literature-proposed management of noncavitated carious lesions [12]. A summary of several studies on noninvasive, microinvasive, and minimally invasive carious lesion management is presented in Table 5. Consequently, the following conclusions are reached:

1. Educating patients on proper oral hygiene and diet habits is critical to avoid or halt the progression of dental caries.
2. Noncavitated proximal lesions were stopped more effectively with infiltration and sealing than noninvasive treatments.
3. Infiltration outperforms sealing in slowing the progression of noncavitated caries lesions.

### 7.4. Step-Wise (SW) Caries Removal (Excavation) Technique

The procedure involves two independent sessions spaced six months apart to allow changes in the dentine and pulp to take place [1, 19, 43, 66]. It is indicated when the carious lesion is close to the pulp radiographically (about 75% into the dentine). The rationale behind the SW caries removal technique, is that partial caries removal (PCR) followed by tooth sealing will result in the lesions being arrested. Furthermore, the counts of anaerobic and aerobic bacteria, *Lactobacilli*, and *Streptococci mutans* would have decreased significantly by the end of treatment [44]. Therefore, caries control does not necessitate complete dentinal caries removal [44], as mentioned earlier. The SW technique is a viable treatment option irrespective of patient age, though it may be more successful in younger patients [66].

In the first visit, the selective caries removal to soft dentine approach is used, and the tooth is then restored with glass ionomer restoration. In the second appointment, 6 to 12 months later, a fresh periapical radiograph, to evaluate periapical pathosis, should be taken. Any signs or symptoms of a possible pulp pathosis should be evaluated, and a sensibility/vitality test must be performed. Selective removal to firm/hard, dry dentine is carried out centrally, or glass ionomer may be used as a base with no additional tissue removal, followed by a composite resin restoration [10].

The SW technique can also be used successfully with a calcium hydroxide-containing base material and a temporary filling [22, 67].

In contrast, some clinical studies have reported promising results in which carious dentine was left in deep cavities, and the cavities were restored with final restoration without re-entry [68, 69].

### 7.5. Indirect Pulp Capping

Indirect pulp capping (IPC) is considered as a selective caries removal to soft dentine [1, 3, 70]. The IPC approach is usually used in deep cavity preparations with or without residual carious dentine that is near to the pulp but does not display apparent pulp exposure [70, 71]. It promotes reparative dentine formation by using material over sound or carious dentine [72]. Hence, it is an example of a selective carious removal method as soft caries dentine is selectively removed [1, 3]. It aims to preserve the vitality of the pulp by selectively removing the caries soft dentin followed by the placement of a therapeutic material such as calcium hydroxide. Calcium hydroxide is traditionally used as a liner, followed by a permanent filling material. However, the use of calcium hydroxide has been questioned due to various drawbacks; as a result, it has been replaced by other biomaterials such as calcium silicate-based materials [71, 73].

This method can be carried out in one or two steps. The final restoration can be placed in the same visit when the one-step method is used. If necessary, a second appointment is scheduled after 6–8 weeks.

### 7.6. Atraumatic Restorative Treatment

It was developed in the mid-eighties to treat caries in children living in economically underdeveloped areas with limited resources and amenities such as electricity and professional staffing [30, 54, 74]. Atraumatic restorative treatment (ART) is a minimally invasive technique that involves the removal of decayed tissue with hand instruments alone, usually without the use of anaesthesia or electrically powered equipment, and the restoration of the dental cavity with glass ionomer cement or resin-modified glass-ionomer cement and compomers [75]. It consists of two clinical steps that are performed at the same clinical appointment. In the first step, soft caries dentine is removed with hand instruments and then restored with high-viscosity glass ionomer restorative material. The nearby pits and fissures are sealed with the same material in the second step. Hence, a high-viscosity restorative glass ionomer fills the cavity and is pushed into the adjacent pits and fissures using the “press-finger” technique. Other restorative materials were also used in a subsequent version of the original procedure [30].

The ART is a valuable therapeutic technique, especially in children, anxious patients, and those with special needs, living in housing for older people, in remote areas or under-resourced communities and the out-reach environment when the appropriate dental instruments and equipment are not available [30]. The advantages of ART include the preservation of tooth structure and the absence of the need for a local anaesthetic, resulting in less discomfort than other standard treatments. The effectiveness of ART, on the other hand, is governed by a variety of factors, including the prevalence of caries, the material used, and the operator's experience [17].

### 7.7. Preventive Resin Restoration

It is also known as conservative composite restoration (CCR). Preventative resin restoration (PRR) is a minimally invasive method that is usually indicated for restoring small carious lesions in the posterior teeth [76]. It involves the removal of caries in one stage [76, 77]. Only caries affected pits and fissures are prepared to receive the filling. The pit and fissure caries are removed, and composite resin is used as a permanent restoration. Glass ionomer may be used as a liner when the carious lesion reaches the dentine. When the resultant cavity is narrow, a flowable resin is usually used [78]. The remaining fissures are then etched and sealed with a fissure sealant material.

## 8. Discussion

Prevention is always better than cure. It is critical to have a strategy or procedures to combat caries before it occurs. A system that focuses on community education programmes would be highly beneficial [47, 79].

The most current strategy for controlling dental caries is an evidence-based approach focused on risk assessment and disease prevention [22]. As a result, a shift from comprehensive eradication of carious lesions to a selective caries removal concept and a minimally invasive or even nonrestorative model has been advocated in recent years [80].

The minimum intervention method is based on a preventative strategy and customized risk assessments; as a result, each patient's condition is handled and managed in accordance with his or her level of risk. Therefore, reliable, early diagnosis of lesions and remineralization of noncavitated lesions are essential, and when operational intervention is required, the approach utilized should be as minimally invasive as possible. Also, bringing the patient into the re-restorative-restorative cycle and replacing poor restorations should be avoided. As a result, if feasible, defective restorations should be polished, refurbished, or repaired.

With regular check-ups, the prevention and management of demineralized lesions and early caries lesions may be achieved with remarkable success using less invasive and more conservative methods. The caries process can be controlled by mechanical plaque control, effective oral hygiene, fluoride and antimicrobial application, and avoiding sugar intake between meals [6, 7]. Resin infiltration of WSLs and sealing of noncavitated lesions as a treatment option have been supported by several studies [51, 56, 59]. This method represents the minimal invasive option.

The nonrestorative cavity control (NRCC) idea is widely employed and has a high success rate [6, 7]. This way carious lesion can be made cleanable by enlarging their openings.

The implementation of selective caries removal has led to a decrease in pulp exposure associated with the nonselective caries removal approach.

The selective caries removal method is based on the decay's proximity to the pulp [3, 5, 7, 39]. As a result, soft dentine may be removed or left in place to avoid pulp exposure.

## 9. Conclusions

It is critical to have a strategy and a system that focuses on community education programmes and patient-centred care must be at the core of our dental responsibilities. Caries management must be based on a caries risk assessment method that is backed up by evidence. The newly available information, knowledge, and materials should encourage professionals to implement this method.

Approaches such as noninvasive, microinvasive, and minimally-invasive should be considered, especially when the carious lesions are not cavitated. The selective caries removal approach is a viable option for preventing caries progression, but careful case selection is required to achieve a good outcome.

Restorative procedures should be minimally invasive to preserve healthy tooth structure and provide long-term restoration. Furthermore, repair or refurbishing should be used wherever possible rather than replacing a defective restoration.

## Figures and Tables

**Table 1:** The features of soft dentine.

(i) Loaded with a high volume of bacteria (ii) Has low mineral content (demineralized) (iii) Has irreversibly denatured collagen (iv) Histologically, it may be referred to as necrotic and contaminated (v) Clinically, it can be easily excavated with hand and rotary instrumentation (vi) It may be retained in extremely deep carious lesions when the possibility of pulp exposure is high

**Table 2:** The characteristics of the firm carious dentine.

(i) Characterised by demineralisation of intertubular dentine (ii) Initial formation of intratubular fine crystals at the advancing front of the caries lesion (iii) The tubule lumen becomes filled with minerals (iv) Histologically, firm dentine may be referred to as demineralized (v) Due to the demineralisation process, firm dentine is softer than hard dentine (vi) Clinically, unlike soft dentine, firm dentine is resistant to hand excavation and can only be removed by exerting some pressure

**Table 3:** A summary of the CAMBRA guideline.

Caries management by risk assessment (CAMBRA) is used to produce a custom risk assessment for individual patients [48]
(1) Assess risk indicators
(2) Assess risk factors
(3) Assess protective factors
(1) Risk indicators
(i) The “WREC” (an acronym for risk indicator factors). These factors consist of the followings
(a) White spot lesions
(b) Restorations (in last 3 years)
(c) Enamel lesions visible on radiographs
(d) Cavitation into dentine
(2) Risk factors
(i) BAD (an acronym for risk factors). These factors consist of the followings
(a) Bacteria (*Streptococcus mutans* and *Lactobacilli* 103–105 cfu)
(b) Absence of saliva
(c) Dietary habits (frequency of snacking with sugary foods; poor oral hygiene)
(3) Protective factors
(i) SAFE (is an acronym for protective factors). These factors include
(a) Saliva and Sealants
(b) Antibacterial (chlorohexidine)
(c) Fluoride
(d) Effective diet/lifestyle habits (including plaque control; use of xylitol)

**Table 4:** Summary of management of noncavitated proximal carious lesions with the most conservative methods.

Noninvasive strategies	Microinvasive strategies	Minimally invasive (restorative) strategies
(i) Based on the use of topical fluorides and other chemical agents to control plaque accumulation, such as interdental cleaning using interproximal brush and floss, rather than removing dental hard tissue, as well as patient education and diet control. (ii) This may be sufficient for lesion arrest in individuals with low caries risk/susceptibility and when lesions are radiographically confined to the enamel.	(i) This represents the dental hard tissue surface removal at the micron level, usually during an acid etching step, such as in sealing or infiltration techniques. The infiltration technique involves etching with 15% HCl-gel for two minutes and then infiltrating with a low-viscositylight-curing resin such as “Resin Infiltration”; Icon; (DMG). (ii) It is recommended for individuals who are at high risk/susceptible or when lesions extend radiographically into dentine. When such treatment is intended, many factors, such as clinical experience or cost, should be considered. (iii) Microinvasive treatment combined with noninvasive measures significantly improves the outcome of noncavitated enamel and initial dentine lesions (limited to the outer third of dentine based on radiograph and clinically noncavitated). It is considerably more efficient than noninvasive management alone. (iv) There is evidence that sealing and resin infiltration can stop lesions confined to the enamel or near the enamel-dentine junction. Still, only infiltration techniques can stop lesions that involve the dentin [52]. (v) The distinction between sealing and infiltration is that while fissure sealing acts as a diffusion barrier on the lesion's surface, infiltration creates a barrier within the lesion by replacing the mineral lost with the resin.	(i) This method entails removing a small amount of dental hard tissue with sharp excavators or rotary instruments. It is usually followed by the replacement of the removed hard tissue with appropriate restorative materials such as composite resin. (ii) Cavitated lesions frequently necessitate restorative strategies. Adhesive direct restorations allow for minimally invasive tooth preparations, making them the material of choice for restoring proximal lesions in many cases. Amalgams, on the other hand, have a lower risk of secondary lesions and failure, and because their placement is less technique-sensitive, they may be preferred in more clinically complex scenarios, depending on national policy guidelines, as amalgam is not used in several countries.

**Table 5:** A summary of several studies on noninvasive, microinvasive, and minimally invasive carious lesion management.

Reference	Study type	Materials and methods	Conclusions
Abuchaim et al. [61]	In vivo	The study included 44 adolescents who had bitewing radiographs taken to diagnose caries. The sample included noncavitated lesions extending up to half the thickness of the dentin. After tooth separation, the proximal caries-lesion surfaces in the experimental group (*n* = 33) were sealed with an adhesive. The control group (*n* = 11) was given oral hygiene instructions, including dental floss. After one year, follow-up radiographs were taken and compared to baseline radiographs.	Approximately 22% of the sealed lesions showed reduction, 61% no change, and 16% progressed. The corresponding values for the control lesions were 27%, 36%, and 36%, respectively. Sealing proximal caries lesions was not shown to be superior to lesion monitoring over a year.

Kantovitz et al. [60]	A systematic review	The Cochrane Library, Embase, PubMed, and Web of Science (ISI) databases were searched for papers published between January 1970 and September 2008.	While fissure sealing acts as a diffusion barrier on the lesion's surface, infiltration creates a barrier within the lesion by replacing the mineral lost with a low-viscositylight-curing resin.

Borges et al. [62]	In vivo	Sixty teeth from patients with a high caries risk had noncavitated dentinal occlusal caries. Patients were randomly assigned to one of two groups, each with 30 teeth. Oral hygiene instructions and a fissure sealant were given to the experiment group. Only oral hygiene instructions were given to patients in the control group. Over a 36-month period, clinical and radiographic examinations were used to track caries progression and sealant loss.	At 36 months, the pit and fissure sealant used in this study was shown to be effective in stopping carious lesions.

Ammari et al. [59]	A systematic review and meta-analysis	A thorough search was carried out in the following systematic electronic databases until June 2013: PubMed, Cochrane Library, Scopus, IBI Web of Science, Lilacs, SIGLE, and ClinicalTrials.gov. The study included only controlled clinical trials and randomised controlled clinical trials that evaluated the effectiveness of sealing on noncavitated proximal caries with a minimum follow-up of 12 months.	The findings indicate that sealing noncavitated proximal caries effectively controls proximal caries in the short and medium term. More long-term randomised clinical trials are needed to strengthen this evidence.

de Assuncao et al. [62]	A systematic review	Through November 2013, the MEDLINE/PubMed, LILACS, SciELO, and Scopus databases were searched for relevant publications. Only clinical trials evaluating the ability of noninvasive methods to stop the progression of occlusal noncavitated dentin carious lesions were considered.	Occlusal fissure sealing with a resin-based sealant may be used to arrest the progression of noncavitated occlusal dentine caries. Additional clinical trials with longer follow-up times are needed to enhance scientific evidence.

Dorri et al. [11]	A systematic review	The Cochrane Oral Health Group Trials Register, the Cochrane Central Register of Controlled Trials (CENTRAL), MEDLINE via OVID, EMBASE via OVID, LILACs via BIREME Virtual Health Library, Web of Science conference proceedings, ZETOC conference proceedings, proquest dissertations and theses, ClinicalTrials.gov, OpenGrey, and the World Health Organization (WHO) International Clinical Trials Registry Platform were all searched until December 31, 2014. The metaRegister of controlled trials was searched up to and including October 1, 2014. There were no language or date restrictions in the electronic database searches. The investigation sought to assess the efficacy of microinvasive treatments for managing proximal caries lesions in children and adults with primary and permanent dentition.	According to the available evidence, microinvasive treatment of proximal caries lesions stops noncavitated enamel and initial dentinal lesions (limited to the outer third of dentine, based on radiograph) and is significantly more effective than noninvasive professional treatment (e.g., fluoride varnish) or advice (e.g., to floss).

Anauate-Netto et al. [63]	In vivo	A controlled clinical trial included 23 volunteers with clinically and radiographically noncavitated occlusal caries and caries risk ranging from “low” to “very high.” A total of 86 teeth were randomly assigned to one of two experimental groups: Group one received a commercial pit-and-fissure sealant; while group two received Icon infiltrant (DMG). Over a three-year period, caries progression was monitored using clinical (laser fluorescence caries detection) and radiographic examinations at 12-month intervals. The marginal integrity of the sealing materials was also evaluated.	After three years of clinical evaluation, the infiltrant was effective in preventing caries progression in noncavitated pit-and-fissures, comparable to the conventional sealant. The infiltrant also showed better radiographic results in caries progression at the 3-year evaluation time.

Krois et al. [64]	Systematic review and meta-analysis	Hand searches and cross-referencing were used in addition to searching three electronic databases (MEDLINE, Embase, and Cochrane Central). Randomized controlled trials comparing microinvasive strategies, noninvasive treatment, or placebo for treating proximal carious lesions were included in the study. The primary outcome was the radiographic progression of the lesion. For synthesis, pairwise and Bayesian network meta-analysis, as well as TSA, were used.	Microinvasive (sealing or infiltration) approaches are likely to be more effective than noninvasive approaches for arresting early (noncavitated) proximal lesions.

Abdelaziz et al. [65]	In vivo study	Extracted human posterior teeth with noncavitated proximal carious lesions (ICDAS code 1-2) were cut vertically to obtain two symmetrical lesions. Group: Noninvasive proximal adhesive restoration (NIPAR)—half of the paired lesions' surfaces (*n* = 13) were abraded with metallic strips and etched with 37% H_3_PO_4_ for 120 seconds. Group 2: The infiltration concept technique (ICON)—the other half of the paired lesions' surfaces (*n* = 13) were etched with 15% HCl gel for 120 seconds. Group 1 samples were infiltrated with Scotchbond universal for 180 seconds. Group 2 samples were infiltrated with ICON infiltrant	Noninvasive proximal adhesive restoration allowed for better infiltration of noncavitated proximal carious lesions than ICON. Clinical significance: The combination of infiltration and sealing using noninvasive proximal adhesive restoration (NIPAR) offers a suitable noninvasive treatment option for noncavitated proximal lesions combining the advantages of sealing and infiltration.

Chen et al. [51]	Systematic review	Six electronic databases were searched for published literature, and references were manually searched. Split-mouth randomised controlled trials comparing the efficacy of infiltration/sealing versus noninvasive treatments in proximal lesions were included. The primary outcome was determined by radiographic readings.	Infiltration and sealing were more efficacious than noninvasive treatments for halting noncavitated proximal lesions.

## Data Availability

No data were used to support this study.
